# Regulatory mechanisms of ryanodine receptor/Ca^2+^ release channel revealed by recent advancements in structural studies

**DOI:** 10.1007/s10974-020-09575-6

**Published:** 2020-02-10

**Authors:** Haruo Ogawa, Nagomi Kurebayashi, Toshiko Yamazawa, Takashi Murayama

**Affiliations:** 1 Institute for Quantitative Biosciences, The University of Tokyo, Tokyo, 113-0032 Japan; 2 Department of Pharmacology, Juntendo University School of Medicine, Tokyo, 113-8421 Japan; 3 Department of Molecular Physiology, The Jikei University School of Medicine, Tokyo, 105-8461 Japan

**Keywords:** Excitation–contraction coupling, Ryanodine receptor, Ca^2+^ release channel, Sarcoplasmic reticulum, Skeletal muscle, Structural biology, Molecular dynamics

## Abstract

Ryanodine receptors (RyRs) are huge homotetrameric Ca^2+^ release channels localized to the sarcoplasmic reticulum. RyRs are responsible for the release of Ca^2+^ from the SR during excitation–contraction coupling in striated muscle cells. Recent revolutionary advancements in cryo-electron microscopy have provided a number of near-atomic structures of RyRs, which have enabled us to better understand the architecture of RyRs. Thus, we are now in a new era understanding the gating, regulatory and disease-causing mechanisms of RyRs. Here we review recent advances in the elucidation of the structures of RyRs, especially RyR1 in skeletal muscle, and their mechanisms of regulation by small molecules, associated proteins and disease-causing mutations.

## Introduction

Intracellular Ca^2+^ is a key secondary messenger in a wide variety of biological functions including muscle contraction (Ebashi and Endo 1968). The Ca^2+^ concentration in the cytoplasm is normally kept very low (~ 0.1 μM). In striated muscle cells, when cells are stimulated, Ca^2+^ is rapidly released from intracellular Ca^2+^ stores of the sarcoplasmic reticulum (SR), a process known as excitation–contraction coupling (E–C coupling) and concentration of total Ca^2+^ released in the cytoplasm immediately reaches > 0.1 mM (Baylor et al. 1983; Bers 2001; Baylor and Hollingworth 2012). This release of Ca^2+^ from the SR is predominantly mediated by ryanodine receptors (RyRs), Ca^2+^ release channels in the SR membrane (Fill and Copello 2002; Bers 2004). RyRs constitute homotetramer and are known as the largest ion channels identified to date. The total molecular weight is ~ 2.2 MDa with each monomer consisting of ~ 5000 amino acid residues. RyRs belong to a member of P-type channel, with six transmembrane (TM) regions at their C-terminus and the large N-terminal cytoplasmic region (Clarke and Hendrickson 2016; Zalk and Marks 2017). RyRs form a fourfold symmetric mushroom-like structure, known as a "foot" as observed in the electron microscopy (Franzini-Armstrong 1973; Franzini-Armstrong and Jorgensen 1994). Three types of isoforms are known for RyR in vertebrates: RyR1 is predominantly expressed in skeletal muscle, RyR2 is predominantly present in cardiac muscle, and RyR3 is ubiquitously expressed in a minuscule amount (Sorrentino 1995; Sutko and Airey 1996).

The primary triggering for the opening of RyRs is an elevation of cytoplasmic Ca^2+^ concentration. Binding of Ca^2+^ to RyRs opens the channel, which is known as Ca^2+^-induced Ca^2+^ release (CICR) (Endo 2009). All RyR isoforms mediate CICR. In cardiac muscle, CICR is considered to be the physiological mechanism of Ca^2+^ release (Fabiato and Fabiato 1978; Nabauer et al. 1989). By contrast, in skeletal muscle, CICR is not the primary mechanism of physiological Ca^2+^ release, although it was initially identified in skeletal muscle (Endo et al. 1970; Ford and Podolsky 1970). RyR1 mediates depolarization-induced Ca^2+^ release (DICR), which is gated via physical association with L-type voltage-dependent Ca^2+^ channels (DHPR, dihydropyridine receptor) (Adams et al. 1990; Rios and Pizarro 1991). In DICR, an increase in Ca^2+^ concentration is not necessary to open the channel. DICR is mediated by RyR1 but not by RyR2 or RyR3 (Yamazawa et al. 1996; Nakai et al. 1997; Fessenden et al. 2000).

There are many reported regulatory molecules of RyRs. Such regulators include from small molecules (e.g., Ca^2+^, Mg^2+^, ATP and caffeine) and proteins [e.g., FK506-binding protein (FKBP) and calmodulin (CaM)] (Meissner 1994; Ogawa 1994). In addition, RyRs are modulated by various posttranslational modifications, including phosphorylation, oxidation, and *S*-nitrosylation (Lanner et al. 2010; Kakizawa et al. 2012). RyRs have been implicated in a number of diseases. Mutations in RyR1 are associated with various muscle diseases, such as malignant hyperthermia (MH), central core disease (CCD), and multi-minicore disease (MmD) (Robinson et al. 2006; Treves et al. 2008) and those in RyR2 are linked with several arrhythmogenic heart diseases, such as catecholaminergic polymorphic ventricular tachycardia (CPVT) (Priori et al. 2002; Tester et al. 2004; Kawamura et al. 2013). A considerable number of disease-associated mutations are localized in the N-terminal domains (NTD) of the cytoplasm despite the distance between the NTD and TM region being more than 120 Å (Tung et al. 2010). The mechanism by which these mutations cause abnormalities in the opening and closing of the channels in the transmembrane (TM) region remains largely unknown.

Recent revolutionary advancements in cryo-electron microscopy (cryo-EM) provides a number of near-atomic protein structures. Although the achievable resolution by cryo-EM depends on the molecular weight of the sample, there are several reported structures with resolution better than 2 Å (Merk et al. 2016). At this resolution, most of side-chains of the amino-acid residues are clearly visible and many water molecules can be identified. The achievable resolution by X-ray crystallography is still higher than that by cryo-EM, and there are some high resolution structures better than 1 Å resolution (Hirano et al. 2016). In fact, at this resolution, information of the positions of hydrogen atoms, distributions of valence electrons and orientations of bound waters can be identified. However, the great advantage with cryo-EM is that there is no need crystallization. As the result, number of near-atomic structures of RyRs, and these structures have increased our understanding of the architecture of RyRs. Thus, we are now beginning to understand the mechanisms of activation (CICR and DICR), regulation and modification of RyRs by regulatory molecules and alterations by disease-causing mutations.

## Overall structure of RyR1—architecture of the channel

Structures of RyRs have been studied extensively by cryo-EM due to the huge size of these channels. Numerous papers have been reported, which include comparison of structures between closed and open states (Samso et al. 2009) and the identification of specific domains (Zhang et al. 2003) and binding sites for ligands (Wagenknecht et al. 1997; Samso et al. 1999). However, since the maximum resolution was limited to ~ 10 Å until 2015 (Ludtke et al. 2005; Samso et al. 2005, 2009), it has been difficult to assess the structure of RyRs at the amino-acid level. X-ray crystallography is one of the most powerful techniques for obtaining high-resolution structures, but the crystallization of such a huge membrane protein is difficult. Thus, the most reasonable approach prior to 2015 was X-ray crystallography of various domains in the large cytoplasmic region (Amador et al. 2009; Lobo and Van Petegem 2009; Tung et al. 2010; Sharma et al. 2012; Kimlicka et al. 2013a, b; Borko et al. 2014; Lau and Van Petegem 2014; Yuchi et al. 2015). In fact, Van Petegem and colleagues were succeeded in superposing the structure of the NTD to the cryo-EM density maps at ~ 10 Å resolution (Tung et al. 2010) and predicting how changes in intermolecular contacts affected gating (Kimlicka et al. 2013a).

Recent advances in the structural analysis by cryo-EM have initiated a "resolution revolution" in the field of structural biology (Subramaniam et al. 2016) and as a result, three different groups reported near-atomic structures of RyR1 in 2015 (Efremov et al. 2015; Yan et al. 2015; Zalk et al. 2015). Other studies have reported numerous structures, including RyR1 in the open state (Bai et al. 2016; Wei et al. 2016); RyR1 with bound small regulatory molecules, such as Ca^2+^, ATP and caffeine (des Georges et al. 2016); RyR2 in the closed and open states (Peng et al. 2016); RyR2 with bound calmodulin (Gong et al. 2019); RyR2 with bound FKBP12.6 (Chi et al. 2019). The list of these near-atomic structures is shown in Table 1.

**Table 1:** Near-atomic structures of RyRs published since 2015

Paper	Condition (state)	Resolution (Å)	PDB ID
RyR1			
Yan et al. (2015)	2 mM EGTA/FKBP12.6 (closed)	3.8	3J8H
Zalk et al. (2015)	2 mM EGTA (closed)	4.8	3J8E
Efremov et al. (2015)	1 mM EGTA (closed)	6.1	4UWA
	10 mM Ca^2+^ (inactivated?)	8.5	4UWE
Bai et al. (2016)	2 mM EGTA/FKBP12 (closed)	3.8–4.2	5GKY, 5GKZ, 5GL0
	50 μM Ca^2+^/10 μM PCB95/FKBP12 (open)	5.7	5GL1
Wei et al. (2016)	100 μM Ca^2+^/10 μM ruthenium red (open)	4.9	5J8V
des Georges et al. (2016)	2 mM EGTA/FKBP12 (closed)	4.4	5TB0, 5TB1, 5TB2, 5TB3, 5TB4
	2 mM ATP/5 mM caffeine/30 μM Ca^2+^/FKBP12 (open)	4.3–4.4	5T9V, 5TA3, 5TAL, 5TAN, 5TAM, 5TAQ
	2 mM EGTA/2 mM ATP/5 mM caffeine/FKBP12 (closed)	4.6	5TAP, 5TAS, 5TAT, 5TAU, 5TAV
	30 μM Ca^2+^/FKBP12 (priming state)	3.8	5T15, 5T9M, 5T9N, 5T9R, 5T9S
	300 μM Ca^2+^/10 μM ryanodine/FKBP12 (open)	4.4	5TAW, 5TAX, 5TAY, 5TAZ
RyR2			
Peng et al. (2016)	5 mM EGTA (closed)	4.2	5GO9
	20 μM Ca^2+^/20 μM PCB95 (open)	4.1	5GOA
Gong et al. (2019)	CaM/FKBP12.6 (apo-CaM)	3.6	6JI8
	5 mM ATP/ 5 mM caffeine/20 μM Ca^2+^/CaM-M/FKBP12.6 (apo-CaM)	3.7–4.2	6JRS, 6JII
	5 mM ATP/5 mM caffeine/20 μM Ca^2+^/FKBP12.6 (Ca-CaM)	3.9–4.2	6JRR, 6JI0
	5 mM ATP/5 mM caffeine/5 mM Ca^2+^/FKBP12.6 (high Ca-CaM)	3.9	6JIY
	20 μM Ca^2+^/20 μM PCB95/CaM/FKBP12.6 (Ca-CaM)	4.4	6JV2
Chi et al. (2019)	20 μM Ca^2+^	6.1	6JG3
	FKBP12.6/20 μM Ca^2+^/10 μM PCB95/FKBP12.6	4.6	6JGZ
	FKBP12.6/20 μM Ca^2+^/5 mM ATP/FKBP12.6	4.8	6JH6
	FKBP12.6/20 μM Ca^2+^/5 mM caffeine/FKBP12.6	4.5	6JHN

**Fig. 1:**
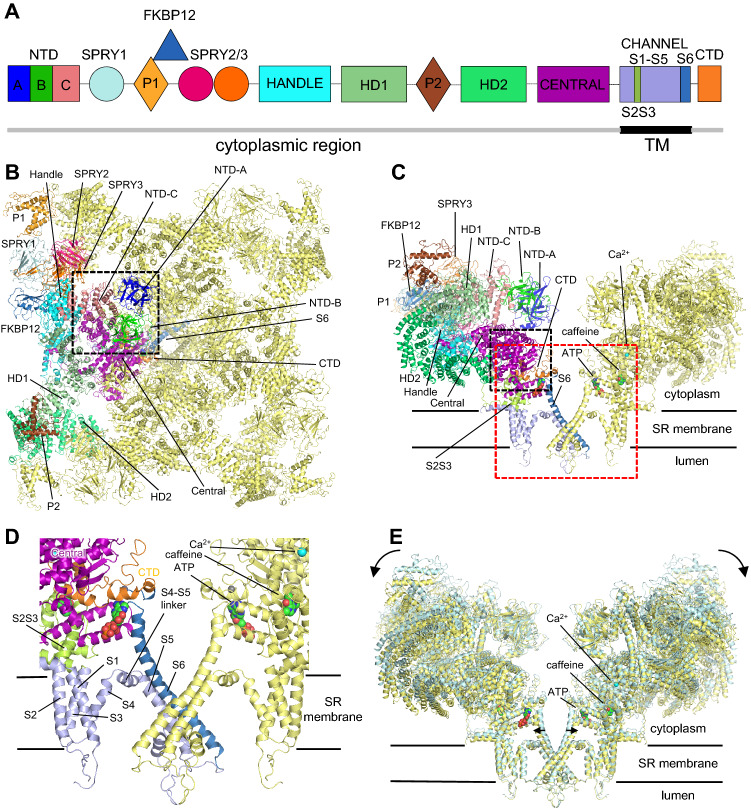
Overall structure and domain organization of the rabbit RyR1. **a** Schematic illustration of domain organization in one protomer. **b** Overall structure of RyR1 in the open state in complex with FKBP12, Ca^2+^, ATP and caffeine looking from the cytoplasmic side. One of four protomers is colored according to the schematic illustration shown in **a** The black dotted square is the area used for the close-up view shown in Fig. 4b. **c** Structure of RyR1 looking from parallel to the membrane. Two of the four protomers facing each other are shown. Ca^2+^ is shown as a cyan ball; ATP and caffeine are shown in sphere representation. The black dotted square is the area used for the close-up view shown in Fig. 4b. **d** Close-up view of the red dotted square shown in **c**. **e** Superimposition of RyR1 in the closed state (light blue) and open state (yellow). Two of the four protomers facing each other are shown. (Color figure online)

Figure 1a shows domain structures of RyR1. Regarding domain notation, different nomenclature has been used by Yan’s group at Tsinghua University and Marks’ group at Columbia University. To avoid confusion, this review will basically follow the domain nomenclature described by Yan’s group. Cytoplasmic domains consist of 12 domains, including 10 N-terminal domains (NTD, SPRY1, P1, SPRY2. SPTY3, Handle, Helical domain 1 (HD1), P2, Helical domain 2 (HD2), and Central domain) before the TM region; the S2S3 region in the TM region; the C-terminal domain (CTD) after the TM region. In addition, a regulatory protein, FKBP12, binds to the cytoplasmic region surrounded by the NTD-C, SPRY1, SPRY3, and Handle domains. The functions and features of each domain are as follows. NTD (N-Terminal Domains) consists of three subdomains: A, B and C. It is one of the hotspots of MH in RyR1 (Lanner et al. 2010). SPRY1/2 constitutes the FKBP12 (FK506 Binding Protein12) binding site (Yuchi et al. 2015). P1 domain locates between SPRY1 and SPRY2 domains also contains MH mutation sites. HD1/2 (Helical Domain1/2) are composed of α-solenoid structure. P2 is one of the phosphorylation sites and is particularly important for regulating activity of RyR2 (Haji-Ghassemi et al. 2019). Central domain is involved in Ca^2+^-binding. Handle domain is important for inducing pronounced structural changes in the Central domain. TM region constitutes ion channel. CTD has a zinc finger and contains one Zn^2+^ in the domain and is also involved in Ca^2+^-binding. Figure 1b, c show the overall structure of RyR1 in the open state with bound Ca^2+^, ATP and caffeine (des Georges et al. 2016) (PDB accession code of 5TAL), looking from the cytoplasmic side and from parallel to the SR membrane, respectively. Figure 1d shows a close-up view of the red dashed boxed area. Interestingly, most domains are tightly linked with each other even though the primary structures are far away (e.g., Handle and Central or Central and CTD). This may be the key to enabling the transmission of motion between distant domains. The black dashed boxed area indicates the binding sites for the regulatory molecules, such as Ca^2+^, ATP, and caffeine, which will be extensively described in the next section. Figure 1e shows the superimposition of RyR1 in the closed and open states. Upon binding of three ligands (Ca^2+^, ATP and caffeine), the huge mushroom-like structure rotates according to the arrow shown in the Fig. 1e. TM helix S6, composing the channel pore, tilts towards the outside of the pore so that the channel pore opens. There is no other major movement of TM helices, except for the S4–S5 linker, which moves towards the outside of the channel pore to make space for the tilting of S6. Basically the same structural changes occur in the structures in the closed and open states of RyR2 (Peng et al. 2016; Chi et al. 2019; Gong et al. 2019) and thus, the underlying mechanisms of the pore opening in RyR1 and RyR2 are considered to be the same. It should be noted that these open-state structures of RyR1 and RyR2 in the open state contain PCB95 (Peng et al. 2016; Gong et al. 2019), ruthenium red (Wei et al. 2016) or ATP plus caffeine (des Georges et al. 2016); Gong et al. 2019) in addition to Ca^2+^. Thus, resolving the Ca^2+^-induced conformational changes that occur in CICR is currently difficult based on these structures. A structure in the open state with only Ca^2+^ bound will be necessary for a complete understanding of the gating mechanism of CICR, although Ca^2+^ alone only partially activates the channel under physiological condition.

## Binding sites for small molecules

**Fig. 2:**
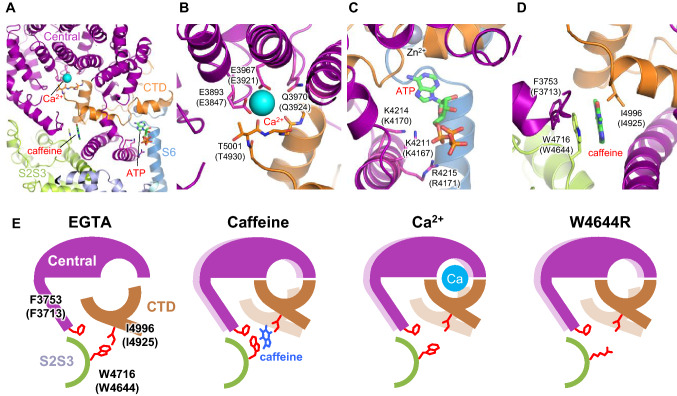
Close-up views of small ligand binding sites. **a** Close-up view of the black dotted square shown in Fig. 1c. **b** Ca^2+^-binding site. **c** ATP-binding site. **d** Caffeine-binding site. Side chains involved in ligands binding are represented as a stick model. Ca^2+^ is shown as cyan ball; ATP and caffeine are shown in stick representation. **e** Schematic diagram of the conformational changes of Ca^2+^-binding and caffeine-binding sites in RyR1. In the presence of EGTA, W4716 in S2S3 and I4996 in CTD interact to pull the CTD towards S2S3 domain, which makes the Ca^2+^-binding pocket larger and less favorable for Ca^2+^ binding. Caffeine breaks the interaction by rotating the tryptophan side chain. This moves the CTD towards Central domain to make the Ca^2+^-binding pocket smaller and more favorable for Ca^2+^ binding. Similar conformational changes occur in response to Ca^2+^ (right most). Hypothetical mechanism by which RyR2-W4644R (RyR1-W4716), a CPVT-associated mutant, causes enhanced Ca^2+^ sensitivity. Mutation in tryptophan (RyR2-W4644R) may also break the interaction to cause an upward shift of the CTD, resulting in enhanced Ca^2+^ sensitivity. Modified from Murayama et al. (2018b). (Color figure online)

Binding sites for three small activating molecules (Ca^2+^, ATP and caffeine) have been identified in RyR1 (des Georges et al. 2016) (Fig. 2a). Interestingly, each ligand binds to the domain interface (Fig. 2a). The Ca^2+^-binding site is located at the interface between Central and CTD; the ATP binding site is located at the interface between Central, S6 and CTD; the caffeine-binding site is located at the interface between Central, S2S3 and CTD. These ligands-binding sites are essentially the same as those identified in RyR2 (Gong et al. 2019). The binding site for ryanodine has been identified around the constriction site of the channel pore, but the atomic model of ryanodine has not yet been built due to poor density (des Georges et al. 2016). Although the addition of PCB95 to RyRs induces the open state, there is no density indicating PCB95 in the density map and its binding sites are still unknown (Bai et al. 2016; Peng et al. 2016).

## Ca^2+^-binding site

RyRs exhibit biphasic Ca^2+^ dependence. They are activated by µM concentrations of Ca^2+^ are inactivated by sub-mM or higer Ca^2+^ concentrations (Meissner 1994; Ogawa 1994). This is explained by the presence of two distinct Ca^2+^-binding sites—high-affinity activating sites and low-affinity Ca^2+^-inactivating sites. The Ca^2+^ concentration for inactivation differs between RyR1 and RyR2; RyR2 requires 10- to 100-fold higher Ca^2+^ than RyR1 (Laver et al. 1995; Laver and Lamb 1998; Murayama and Kurebayashi 2011). The putative high-affinity Ca^2+^-binding site for channel activation in RyR1 has been proposed to consist of two glutamate residues (RyR1-E3893 and RyR1-E3967) in Central domain and one carbonyl oxygen atom (T5001O) in CTD (des Georges et al. 2016) (Fig. 2a, b). These residues are all conserved in RyR2 as RyR2-E3847, RyR2-E3921 and RyR2-T4930O, respectively. According to the proposal, two groups performed functional assay using RyR1 or RyR2 carrying mutations at the putative Ca^2+^-binding site (Murayama et al. 2018b; Xu et al. 2018). Since mutations at residues involved in Ca^2+^-binding reduce the affinity for Ca^2+^, the mutant channels are expected to have reduced or lost Ca^2+^ sensitivity. Murayama et al. created alanine mutants (Murayama et al. 2018b) and Xu et al. created glutamine or valine mutants of the residues (Xu et al. 2018). These mutants completely lost Ca^2+^-dependent activation. They concluded that the putative Ca^2+^-binding site in the cryo-EM structure is the actual Ca^2+^-binding site. Interestingly, Murayama et al. also created additional aspartate mutants of the two glutamates, and found that RyR2-E3921D (RyR1-E3967D) exhibited similar Ca^2+^ sensitivity to the WT, while RyR2-E3847D (RyR1-E3893D) showed only minimal binding at higher Ca^2+^ concentrations. This suggests that RyR1-E3967 can more flexibly coordinate with Ca^2+^ than RyR1-E3893. This is supported by the structure, showing that the side chain of RyR1-E3893 has an extremely bent structure towards the bound Ca^2+^ (Fig. 2b).

It has been proposed that RyR1-Q3970, near the Ca^2+^-binding site, may be directly involved in the direct binding to Ca^2+^ (des Georges et al. 2016). However, in the current structure, the distance between the Oε of the asparagine residue and Ca^2+^ in the structure of RyR1 is long (4.3 Å) and seems to be unsuitable for the coordination of Ca^2+^ binding. Nevertheless, RyR1-Q3970 and RyR2-Q3925 are very important residues, since RyR1-Q3970K causes central core disease (Snoeck et al. 2015) and RyR2-Q3925E is associated with arrhythmogenic syndrome (Medeiros-Domingo et al. 2009). Indeed, Chirasani et al. (2019) demonstrated that RyR1-Q3970K and RyR2-Q3925E exhibit low Ca^2+^-dependent channel activity and Murayama et al. found that RyR2-Q3924A/E mutants (RyR1-Q3970A/E) have reduced Ca^2+^ sensitivity (Murayama et al. 2018b). Taken together, these results indicate that RyR1-Q3970 (RyR2-Q3924) is not directly but rather, indirectly involved in the Ca^2+^ binding.

It is surprising that the number of oxygen atoms coordinating Ca^2+^ binding in RyR1 and RyR2 is only 3, since 6 to 7 coordinating oxygen atoms are usually required for high-affinity Ca^2+^-binding sites (Nayal and Di Cera 1994). The most likely explanation for this is that water molecules that could not be resolved in the current resolution and they may be involved in the coordination of Ca^2+^ binding. Structures with a resolution high enough to resolve water molecules are needed to answer this question.

Another unanswered question is in relation to low-affinity Ca^2+^-inactivating sites. Since RyRs are inactivated by sub-mM or more Ca^2+^ concentrations (Meissner 1994; Ogawa 1994), low-affinity Ca^2+^-binding sites should exist in RyRs and they may be responsible for the Ca^2+^-dependent inactivation. The identification of these sites using functional studies is progressing. Gomez et al. used an RyR1/RyR2 chimera to demonstrate that two different regions in RyR1 (EF-hand-type Ca^2+^-binding motif in Central domain and the S2S3 region) are involved in Ca^2+^-dependent inactivation, through direct or indirect mechanisms (Gomez and Yamaguchi 2014). Since these two regions are very close in the RyR1 structure (Yamaguchi 2020), it is possible that they compose the low-affinity Ca^2+^-binding site. Gomez et al. also demonstrated that MH-associated mutations in the S2S3 region greatly reduce Ca^2+^-dependent inactivation (Gomez et al. 2016). This may result in a gain-of-function of RyR1, thus leading to MH. In the current RyR1 structures, no Ca^2+^, other than that bound at the high-affinity Ca^2+^-binding site, has been resolved. Observations with sub-mM and 10-mM levels of Ca^2+^ for RyR1 and RyR2, respectively, are needed to identify the low-affinity Ca^2+^-binding site.

## ATP-binding site

ATP is known as a stabilizer of the opening of RyRs induced by Ca^2+^. ATP potentiates CICR without altering the dependence of CICR on Ca^2+^ concentration (Meissner 1994). The binding site for ATP in RyR1 is located at the interface between Central domain, S6 and CTD (des Georges et al. 2016) (Fig. 2a, c). The adenine ring of ATP is positioned exactly in the pocket composed of the interface between S6 and CTD (Fig. 2c). The β- and γ-phosphates of ATP bound to RyR1 protrude from the pocket and form contacts with positively charged residues (RyR1-K4211, RyR1-K4214 and RyR1-R4215) in Central domain (Fig. 2c). Both phosphates likely to form individual bonds with the positively charged residues. Therefore, it is thought that ATP has two distinct roles—exact occupation of the pocket composed of the interface between S6 and CTD by the adenine ring; binding of the triphosphate protruding from the pocket to Central domain. These results may explain the following two questions: (i) why adenine-based nucleotides function as activators of RyRs, while other non-adenine nucleotides (e.g., GTP, CTP, TTP) do not significantly activate RyRs and (ii) why the extent of the activation is in the order, ATP > ADP > AMP (Meissner 1994). A recent study by Lindsay et al. (2018) also supports the above two distinct roles of ATP. In their study, a combination of adenosine and triphosphate (PPPi) was found to greatly increase potentiating activity, although the PPPi moiety alone was capable of activating RyR2. Interestingly, PPPi produced two distinct effects, activation and irreversible inactivation, most likely by binding to two distinct sites (the PPPi site and the pocket for the adenine ring). Thus, the adenosine moiety may guide PPPi to a suitable position for its activating effects. The mechanism whereby ATP potentiates the channel remains unclear. The interaction between Central domain and CTD may be important for the potentiating effects of ATP. However, it is difficult to understand the structural changes induced by ATP, since there is no structure of RyRs with ATP alone so far. Further studies are required to answer this important question.

## Caffeine-binding site

Caffeine, a xanthine derivative, is a potent and common activator of all known RyR isoforms. It greatly increases the sensitivity of CICR to Ca^2+^ and causes CICR channel opening even at steady-state cytoplasmic Ca^2+^ concentrations (Rousseau et al. 1988). The caffeine-binding site is composed of RyR1-F3753 in Central domain, RyR1-W4716 in S2S3, RyR1-I4996 in CTD (des Georges et al. 2016) (Fig. 2a, d). These residues are also conserved in RyR2 as RyR2-F3715, RyR2-W4646 and RyR2-I4927. It should be noted that CTD is located between the Ca^2+^-binding site and the caffeine-binding site (Fig. 2a). This may indicate that binding of caffeine pushes CTD towards the Ca^2+^-binding site, resulting in an increased affinity for Ca^2+^ and an increase in Ca^2+^ sensitivity. Murayama et al. elegantly explained this increase in Ca^2+^ sensitivity induced by caffeine (Murayama et al. 2018b). They focused on the residue of RyR1-W4716, since mutation of this tryptophan in human RyR2 (W4645 for human, W4644 for mouse) is reported to cause CPVT (Beery et al. 2009), indicating the physiological significance of the residue. Through functional assays with various mutant channels, they proposed the action of caffeine as follows. When caffeine is not bound, RyR1-W4716 forms a tight hydrophobic interaction with RyR1-I4925 to pull down CTD, thus making the Ca^2+^-binding pocket less favorable for Ca^2+^ binding (Fig. 2e, EGTA). Caffeine then binds stably by forming a π-interaction with the indole ring of the RyR1-W4716 (Fig. 2e, Caffeine). Upon binding of caffeine, the tryptophan side chain rotates to break the interaction with the isoleucine and to form an interaction with RyR1-F3753 (Fig. 2e, Caffeine). Through these conformational changes, the Ca^2+^-binding pocket becomes smaller and more favorable for the Ca^2+^ binding (Fig. 2e, Caffeine), which is similar to the state seen with bound Ca^2+^ (Fig. 2e, Ca^2+^). In the tryptophan mutant, the interaction with isoleucine is lost, resulting in an increase in Ca^2+^ sensitivity (Fig. 2e, W4644R). Thus, the hydrophobic interaction negatively regulates Ca^2+^ sensitivity. Murayama et al. also found that two CPVT-associated mutations (RyR2(human)-C4193W and RyR2(human)-A4607P) near the caffeine-binding sites greatly increase Ca^2+^ sensitivity by altering the hydrophobic interaction (Murayama et al. 2018b). Structural studies of RyRs with mutated caffeine-binding sites will confirm these findings. Although no disease-associated mutations have been found in RyR1 near or within the caffeine-binding site so far, it is possible that mutations in these sites may cause MH or MH/CCD.

## Calmodulin-binding sites

**Fig. 3:**
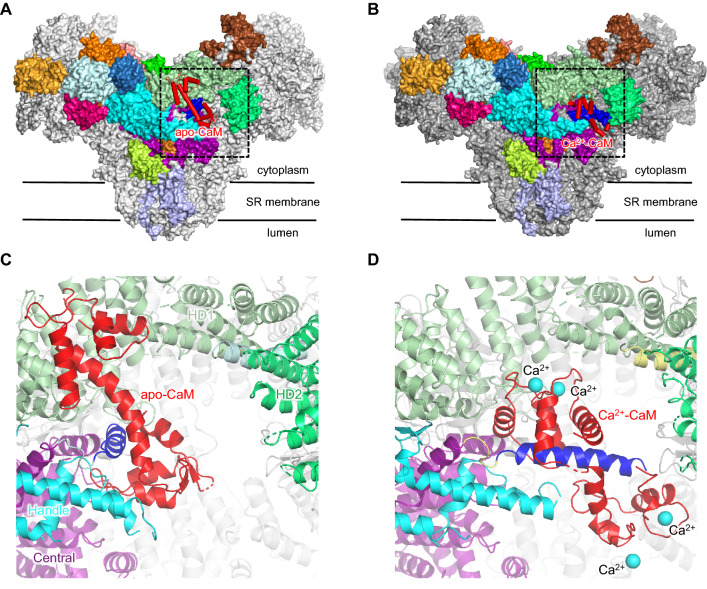
Overall structures of RyR2 in complex with calmodulin. RyR2 in complex with apo-CaM (**a**) and in complex with Ca^2+^-CaM (**b**) are shown as surface representations. One of the four protomers is colored according to the schematic illustration shown in Fig. 1a. Bound CaM is shown as red cylinder models. **c**, **d** Close-up views of the dotted square in **a** and **b**, respectively. All the molecules are represented as ribbon models. Ca^2+^ is shown as a cyan ball. The binding schemes of the apo-CaM and Ca^2+^-CaM to RyR2 are different. CaM in Ca^2+^-CaM bends at the center of the dumbbell and the α-helix (blue colored, amino-acid residues 3585–3668 in RyR2) is surrounded by the Ca^2+^-CaM. As a result, the α-helix is pulled out from the inside of RyR2 and rotates approximately 60 degrees. (Color figure online)

Calmodulin (CaM) is a member of the EF-hand Ca^2+^-binding protein family that regulates Ca^2+^ release from the SR by directly binding to RyRs. Regulation of RyRs by CaM is isoform specific. In the case of RyR1, CaM exhibits biphasic regulation depending on Ca^2+^ concentration. It acts as a weak activator at nanomolar concentrations of Ca^2+^ (apo-CaM) and as an inhibitor at micromolar concentrations of Ca^2+^ (Ca^2+^-CaM) (Tripathy et al. 1995; Balshaw et al. 2001). In the case of RyR2, Ca^2+^-CaM only inhibits the channel, with no activating effects (Fruen et al. 2000; Balshaw et al. 2001). A search for CaM-binding sites revealed that both apo-CaM and Ca^2+^-CaM bind at a single conserved high-affinity site (amino-acids residues 3614–3643 in RyR1 and 3581–3610 in RyR2) (Moore et al. 1999; Yamaguchi et al. 2001). Recently, the structures of RyR2 in complex with apo-CaM and Ca^2+^-CaM at near-atomic resolution have been determined and the molecular mechanism of CaM binding to RyR2 has been clarified (Gong et al. 2019). Indeed, binding sites for apo-CaM and Ca^2+^-CaM overlap in an elongated cleft formed by Handle, HD1 and Central domains (Fig. 3a, b). CaM is a dumbbell-shaped molecule consisting of N- and C-terminal domains, each of which contains two Ca^2+^-binding sites (Babu et al. 1985). In apo-CaM, the upper dumbbell forms contacts with HD1 and the lower dumbbell forms contacts with one of the α-helices in Central domain (blue colored, amino-acid residues 3585–3668 in RyR2) (Fig. 3c). However, in Ca^2+^-CaM, the α-helix (blue colored) is pulled out from inside RyR1 and rotates approximately 60 degrees (Fig. 3b, d). CaM then folds in the middle of the helix connecting the dumbbells and binds to the α-helix (blue colored, Fig. 3d). Interestingly, as predicted by Rodney et al., Ca^2+^ binding to CaM leads to an N-terminal shift in its binding site in the α-helix (Rodney et al. 2001). In fact, in the case of apo-CaM, RyR2-F3604 contacts with F90-CaM and F142-CaM, whereas, in the case of Ca^2+^-CaM, both RyR2-F3604 and RyR2-V3600 are involved in binding to F20-CaM and F69-CaM.

Although structures of RyR2 in complex with CaM have been determined at near-atomic resolution, high resolution structures of RyR1 in complex with CaM have not been reported. Thus, the mechanism of the activating effect of apo-CaM on RyR1 still remain unknown. High-resolution structures of RyR1 with bound CaM are expected to be generated in future research.

## Understanding of disease-causing mechanisms with a view to improved therapy

**Fig. 4:**
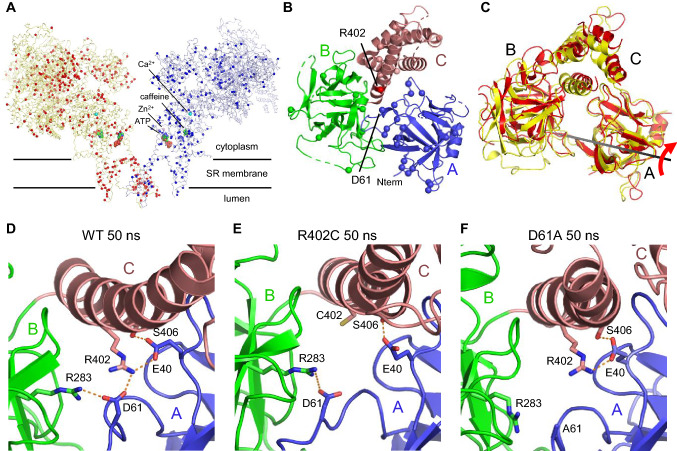
**a**, **b** Mapping of the disease-causing mutations onto the structure RyR1 and RyR2. **a** One of the four protomers of RyR1 and RyR2 is shown as a Cα trace. Left (light yellow) and right (light blue) show RyR1 and RyR2, respectively. Cα atoms of the residues that are targeted for disease-causing mutations are highlighted as red (RyR1) or blue (RyR2) spheres. Ca^2+^ is shown as a cyan ball, Zn^2+^ is shown as a violet ball and ATP and caffeine are shown in sphere representation. **b** Close-up view of the dotted square shown in Fig. 1c. Cα atoms of the residues of disease-causing mutations in the NTD of RyR1 are shown as spheres. R402 that was used for the molecular dynamics (MD) simulation is shown as a red sphere. **c** Superimposition of the monomer of the WT (yellow) and R402C mutant (magenta) after 50 ns of the MD simulation. The BC domains rotated 13.5 ± 0.7 degrees with respect to the A domain in the R402C mutant. **d**–**f** Close-up views around residue 402 after 50 ns of the MD simulation of WT (**d**), R402C (**e**) and D61A (**f**). Colors of domains are the same as shown in Fig. 1a. Dashed lines represent hydrogen bonds/salt bridges. There is a tight network composed of B(R283)-A(D61)-C(R402)-A(E40)-C(S406) (B–A–C–A–C network) around R402, which is critical for the connection of A, B, and C domains. **e**, **f** The same view after 50 ns of MD simulation of the NTD of the disease-causing mutant, R402C and the artificial mutant, D61A. Since C402 and A61 cannot form a hydrogen bond with D61 and R402, respectively, the B–A–C–A–C network is broken. Therefore, the BC domains rotated with respect to the A domain as shown in **c**. **c**–**f** Modified from Yamazawa et al. (2020). (Color figure online)

To date, over 300 mutations have been identified in both RyR1 and RyR2, and these are distributed throughout the molecule (Fig. 4a). NTD is at least 120 Å away from the channel pore, which raises the question of how mutations in the cytoplasmic region affect channel gating. The complete understanding of the disease-causing alterations by mutations requires near-atomic structures of mutant RyRs. However, such structures are currently not available. Several alternative approaches have been taken using the available structures of WT channels. Murayama et al. (2016) performed functional studies of various RyR1 channels carrying disease-associated mutations located in HD1. Evaluation of the three parameters for CICR (Ca^2+^ sensitivity for activation; Ca^2+^ sensitivity for inactivation; attainable maximum activity, i.e., gain) using live-cell Ca^2+^ imaging and [^3^H]ryanodine binding assays revealed qualitative and quantitative differences in alterations between mutations. Then, they observed tertiary structure of RyR1 determined by cryo-EM and the interactions that are important for channel regulation were predicted. Van Petegem et al. used X-ray crystallography of mutant RyR1 channels (Kimlicka et al. 2013a) to determine the atomic structure of the NTD consisting of three domains (A, B, and C) (Tung et al. 2010) (Fig. 4b). By comparing the structures of WT and N-terminal disease-associated mutants, they concluded that mutations affecting inter-domain interactions may alter the relative locations of domains, resulting in altered relative domain orientations between protomers.

Molecular dynamics (MD) simulation is a powerful tool to test how mutations affect the structure. Zheng et al. performed MD simulations of RyR1 or RyR2 mutants using the cryo-EM-determined structure of the NTD tetramer (Zheng and Liu 2017; Xiong et al. 2018). Based on the hydrogen bond analysis after the simulations, they concluded that interactions in the inter-NTD are important. Yamazawa et al. (2020) recently reported MD simulations of the NTD monomer of RyR1, in combination with functional studies. They found that the mutations located around inter-domain region differentially affected hydrogen bonds/salt bridges. In particular, mutations at R402 (R402C/H) were found to cause a clockwise rotation of BC domains with respect to the A domain by altering the inter-domain interactions (Fig. 4c). They also found a hydrogen bonds/salt bridge network between domains (B–A–C–A–C network) that is broken by mutations at R402 (Fig. 4d, e). Importantly, artificial mutants that break the network exhibit activation of the channel in functional analysis and rotation of BC domains in MD simulations. They concluded that inter-domain interactions within the NTD are important for the regulation of RyR1 channel (Fig. 4f).

The discovery of novel compounds that reverse the alterations induced by disease-causing mutations is a promising approach for developing new therapies for RyR1-related diseases. The determination of the structures of RyR1 in complex with these compounds at near-atomic resolution by cryo-EM will identify the binding sites of the compounds and clarify their effects on RyR1, thus allowing a greater understanding of the disease-causing mechanisms. Recently, two groups have developed high-throughput screening methods to discover modulators of RyRs (Rebbeck et al. 2017; Murayama et al. 2018a). Rebbeck et al. (2017) developed a method to monitor time-resolved fluorescence resonance energy transfer (FRET) between FKBP12 and CaM bound to RyR1, which reduces by activation of the channel. Murayama et al. (2018a) monitor Ca^2+^ concentration in the ER of HEK293 cells expressing RyR1, which is reduced by the opening of the channel. These approaches will accelerate the identification of novel RyR modulators.

## Perspectives: toward understanding the mechanism of DICR

In skeletal muscle E–C coupling results in the release of Ca^2+^ from the SR by DICR, which occurs via direct or indirect interactions between DHPR and RyR1 (Rios and Pizarro 1991). DHPR is a 450 kDa hetero-multimeric complex composed of α1 core subunit, auxiliary subunits a2/δ, β and γ subunit (Catterall 2011; Bannister and Beam 2013). It has been shown that the II–III loop of the α1 subunit is responsible for physiological coupling to RyR1 (Tanabe et al. 1990; Lu et al. 1994; El-Hayek et al. 1995; Leong and MacLennan 1998b; Grabner et al. 1999). In contrast, the binding sites for the II–III loop in RyR1 are not clearly defined. Chimeric studies have identified two different regions [residues 1–1680 (Perez et al. 2003) and residues 1635–2636 (Nakai et al. 1998; Proenza et al. 2002)] as the binding site; an in vitro study identified 37 residues (1076–1112) as the binding site (Leong and MacLennan 1998a); NMR chemical shift perturbation analyses and fluorescence studies identified residues 1085–1208, which correspond to SPRY2 domain, as the binding site (Casarotto et al. 2006; Cui et al. 2009); Deletion of 1303–1367 (a part of SPRY3 domain) preserved the function of RyR1 as a Ca^2+^ release channel but resulted in the loss of E–C coupling (Yamazawa et al. 1997). β subunit of DHPR is an indispensable component for DICR (Gregg et al. 1996; Cheng et al. 2005; Schredelseker et al. 2005; Karunasekara et al. 2009). It has been reported that the last 35 residues of the β subunit of DHPR are important for binding to RyR1 (Rebbeck et al. 2011). In vitro binding experiments have identified the binding site for the β subunit of RyR1 as residues 3495–3502, a cluster of positively charged residues (Cheng et al. 2005).

Recently cryo-EM structures of rabbit DHPR have been determined at near-atomic resolution (Wu et al. 2015, 2016; Zhao et al. 2019). All five subunits were resolved and the molecular structure was well understood. However, the required elements for the RyR1 binding such as the II–III loop of the α1 subunit and most of the β subunit including the last 35 residues are missing from the structure. Even in the structure of RyR1, residues 3495–3502 (a part of HD2), which are considered to be part of the binding site of the β subunit of DHPR, are missing (Clarke and Hendrickson 2016; des Georges et al. 2016). Therefore, even the construction of a hypothetical combined model of DHPR and RyR1 is currently difficult. To overcome this situation, one idea may be a reconstitution of DHPR and RyR1, and the determination of the super-complex by cryo-EM. In fact, a recent paper demonstrated that DHPR, STAC3, junctophilin2 (JP2) and RyR1 are the minimum requirements for the reconstitution of conformational coupling (Perni et al. 2017). STAC3 has been identified as an essential protein for E-C coupling and it binds to the II–III loop of the α1 subunit of DHPR. Its structure has already been determined (Wong King Yuen et al. 2017). JP2 is a single-spanning transmembrane protein localized in the SR (Takeshima et al. 2000). It has been reported that N-terminal residues of JP2 bind to the last 12 residues of α subunit of DHPR (Nakada et al. 2018). To form the super-complex, reconstitution of RyR1 and JP2 in a membrane patch, using a nanodisk or a similar tool (Zhang and Cherezov 2019), is required, followed by reconstitution of DHPR on another membrane patch. The super-complex can then be created by mixing both reconstituted proteins with STAC3.

## Conclusion

Since the first three reports of the near-atomic structures of RyR1 in 2015, there have been great advancements in our understanding of the structure–function relationship of RyRs. The binding sites of regulatory small molecules such as Ca^2+^, ATP and caffeine have now been clarified. Moreover, near-atomic structures with bound regulatory proteins such as calmodulin (CaM) have been determined in two different states (apo-CaM and Ca^2+^-CaM), and the molecular mechanism of Ca^2+^-CaM binding to RyR2 has also been clarified. However, this is just the first step in understanding the structure–function relationship of RyRs. In fact, the fundamental mechanisms of RyR, neither CICR nor DICR is still unclear just from the recent structures. It should be noted that the current models of RyRs by cryo-EM have major problems, in that 30% (1500 residues) of the regions of the receptor have no model building completed or amino acid residues identified, especially in the cytoplasmic region (Clarke and Hendrickson 2016). These missing regions include many important functional domains, such as SPRY3, Central, HD2, P1, and P2. There are X-ray crystal structures of such domains, except the NTD (Yuchi et al. 2015), but no structure that spans multiple domains has been reported. Therefore, we still have difficulty in understanding regulatory mechanism that occur via these domains. Finally, all samples currently used for the structural studies by cryo-EM have been extracted and purified from natural sources. However, to understand disease-causing mechanisms, with a view towards developing new therapies, the structures of mutants are indispensable. Determining the structures of recombinant RyRs will be an important focus of future research.
